# Weighted Moments Estimators of the Parameters for the Extreme Value Distribution Based on the Multiply Type II Censored Sample

**DOI:** 10.1155/2013/281624

**Published:** 2013-06-26

**Authors:** Jong-Wuu Wu, Sheau-Chiann Chen, Wen-Chuan Lee, Heng-Yi Lai

**Affiliations:** ^1^Department of Applied Mathematics, National Chiayi University, Chiayi, Taiwan; ^2^Department of International Business, Chang Jung Christian University, Tainan 71101, Taiwan

## Abstract

We propose the weighted moments estimators (WMEs) of the location and scale parameters for the extreme value distribution based on the multiply type II censored sample. Simulated mean squared errors (MSEs) of best linear unbiased estimator (BLUE) and exact MSEs of WMEs are compared to study the behavior of different estimation methods. The results show the best estimator among the WMEs and BLUE under different combinations of censoring schemes.

## 1. Introduction

The lifetime *Y* of a special component has an extreme value distribution with the location parameter *θ* and scale parameter *σ* if its cumulative distribution function (c.d.f.) is given by

(1) $$\begin{array}{c} F_{Y}\left(y\right)=\exp\left[-e^{-(y-\theta )/\sigma }\right],\;-\infty <y<\infty ,\;\;\; \end{array}$$

where −*∞* < *θ* < *∞*, *σ* > 0. If (*Y* − *θ*)/*σ* = *X*, then the c.d.f. of *X* can be written as *F*_*X*_(*x*) = exp⁡[−*e* ^−*x*^], − *∞* < *x* < *∞*. The distribution has been applied in the fields of life testing and reliability [1, 2].

Many researches in estimators of location and scale parameters for the extreme value distribution have been discussed. Balakrishnan and Chan [3] presented tables of the means, variances, and covariances of order statistics from extreme value distribution based on the complete sample. In life testing experiments, the experimenter might not always be in a position to observe the lifetimes of all the items put on test due to time limitations and/or other restrictions (such as dearth of bankroll or material resources). Therefore, multiplying type II censored sample may arise in practice. Let us suppose, for instance, that out of *n* items put on life test, the first *r* lifetimes *Y*_(1)_ < *Y*_(2)_ < ⋯<*Y*_(*r*)_ have only been observed and the lifetimes for the rest (*N* − *r*) components remain unobserved or missing. This type of censoring is known as right type II censoring. Another way to get censored data is to observe the largest *s* lifetimes *Y*_(*N*−*s*+1)_ < *Y*_(*N*−*s*+2)_ < ···<*Y*_(*N*)_. The lifetimes of first (*N* − *s*) components are missing, called left type II censoring scheme. Moreover, if the left and right censoring situations arise together, this is known as doubly type II censoring scheme [4–9]. In addition, a reverse situation to doubly type II censoring is midcensoring where the data on two extremes are available, but some middle observations are censored [10]. However, these censoring schemes are special cases of multiply type II censoring scheme. Furthermore, if the left and right censoring cases arise together, this is known as doubly type II censoring scheme. In addition, a reverse situation to doubly type II censoring is middle censoring where the data on two extremes are available, but some middle observations are censored. However, these censoring schemes are special cases of multiply type II censoring scheme [11, 12].

In this paper, we propose the WMEs of the location parameter *θ* and the scale parameter *σ* from extreme value distribution based on the multiply type II censored sample. The WMEs are derived in Section 2. We also extend the methods of Balakrishnan and Cohen [13] and Balakrishnan and Chan [4] to find BLUEs of the location and scale parameters under multiply type II censored sample. To study the behavior of different estimation methods, simulated MSEs of BLUE and exact MSEs of WMEs are compared and discussed in Section 3. We obtain the best estimator of *θ* and *σ* among the WMEs and BLUE, respectively. Finally, conclusions are given in Section 4.

## 2. The Weighted Moments Estimators and the Best Linear Unbiased Estimator

Suppose that the experimenter fails to observe the first *r*, the last *s*, and the middle *l* observations. Observations *Y*_(*r*+1)_ < *Y*_(*r*+2)_ < ⋯<*Y*_(*r*+*k*)_ < *Y*_(*r*+*k*+*l*+1)_ < *Y*_(*r*+*k*+*l*+2)_ < ⋯<*Y*_(*N*−*s*)_ are the available multiply type II censored sample from (1). To eliminate the effect of the location parameter, we subtract *Y*_(*r*+1)_ from each statistic, and a new available multiply type II censored sample can be written a 0 < *Y*_(*r*+2)_ − *Y*_(*r*+1)_ < ⋯<*Y*_(*r*+*k*)_ − *Y*_(*r*+1)_ < *Y*_(*r*+*k*+*l*+1)_ − *Y*_(*r*+1)_<*Y*_(*r*+*k*+*l*+2)_ − *Y*_(*r*+1)_ < ⋯<*Y*_(*N*−*s*)_ − *Y*_(*r*+1)_. First, by using this censored data, we propose the following WMEs to estimate the scale parameter *σ*:

(2) σ^1=w11[∑i=r+2r+k(Y(i)−Y(r+1))]+w12[∑i=r+k+l+1N−s(Y(i)−Y(r+1))]=w11C11+w12C12,

(3) σ^2=w21[∑i=r+2r+k(Y(i)−Y(r+1))+l(Y(r+k)−Y(r+1))]+w22[∑i=r+k+l+1N−s(Y(i)−Y(r+1))+s(Y(N−s)−Y(r+1))]=w21C21+w22C22,

(4) σ^3=w31[∑i=r+2r+k(Y(i)−Y(r+1))]+w32[∑i=r+k+l+1N−s(Y(i)−Y(r+1))+s(Y(N−s)−Y(r+1))]=w31C31+w32C32,

(5) σ^4=w41[∑i=r+2r+k(Y(i)−Y(r+1))+l(Y(r+k)−Y(r+1))]+w42[∑i=r+k+l+1N−s(Y(i)−Y(r+1))]=w41C41+w42C42.

We apply (2) to (5) to compute their weights by minimizing their MSEs $\underset{w_{i1},w_{i2}}{\min}\text{MSE}\left({\hat{\sigma }}_{i}\right)$, *i* = 1, 2, 3, 4, or equivalently

(6) min⁡wi1,wi2wi12bi1+wi22bi2+2wi1wi2bi3  +(wi1ai1+wi2ai2−1)2, i=1,2,3,4,

where *a*_*ij*_ = *E*(*C*_*ij*_)/*σ*, *b*_*ij*_ = Var⁡(*C*_*ij*_)/*σ* ^2^,  *i* = 1, 2, 3, 4, *j* = 1, 2 and *b*_*i*3_ = Cov(*C*_*i*1_, *C*_*i*2_)/*σ* ^2^, *i* = 1, 2, 3, 4. By using the results of Lieblein [14] and *Y*_(*i*)_ = *θ* + *σX*_(*i*)_, taking *i* = 1 as example, *a*_11_, *a*_12_, *b*_11_, *b*_12_, and *b*_13_ are obtained as follows:

(7) a11=∑i=r+2r+kE(X(i))−(k−1)E(X(r+1)),a12=∑i=r+k+l+1N−sE(X(i))−(N−s−r−k−l)E(X(r+1)),b11=∑i=r+2r+kVar⁡(X(i))+2∑i=r+2r+k−1∑j=i+1r+kCov(X(i),X(j))+(k−1)2Var⁡(X(r+1))−2(k−1)∑i=r+2r+kCov(X(i),X(r+1)),b12=∑i=r+k+l+1N−sVar⁡(X(i))+2∑i=r+k+l+1N−s−1 ∑j=i+1N−sCov(X(i),X(j))+(N−s−r−k−l)2Var⁡(X(r+1))−2(N−s−r−k−l)∑i=r+k+l+1N−sCov(X(i),X(r+1)),b13=∑i=r+2r+k ∑j=r+k+l+1N−sCov(X(i),X(j))−(N−s−r−k−l)∑i=r+2r+kCov(X(i),X(r+1))  −(k−1)∑j=r+k+l+1N−sCov(X(r+1),X(j))+(k−1)(N−s−r−k−l)Var⁡(X(r+1)),

where

(8) E(X(i))=NCi−1N−1∑p=1N−i+1(−1)p−1×Cp−1N−i1i+p−1[γ+log⁡(i+p−1)],Cov(X(i),X(j))  =ijCjNCij−1∑p=1j−i∑q=1N−j+1(−1)p+q−2 ×Cp−1j−i−1Cq−1N−jϕ(i+p−1,j−i−p+q) −{NCi−1N−1∑p=1N−i+1(−1)p−1Cp−1N−i×1i+p−1[γ+log⁡(i+p−1)]} ×{NCj−1N−1∑q=1N−j+1(−1)q−1Cq−1N−j×1j+q−1[γ+log⁡(j+q−1)]},

*γ* = −Γ^′(1) is Euler's constant and *ϕ*(*t*, *u*) = ∫_−*∞*_^*∞*^∫_−*∞*_^*y*^ *xye* ^−*x*−*te*^−*x*^^ *e* ^−*y*−*ue*^−*y*^^ *dx* *dy* [14].

Finally, by differentiating (6) with respect to *w*_*i*1_ and *w*_*i*2_, we obtain

(9) $$\begin{array}{c} w_{i1}=\frac{b_{i2}a_{i1}-b_{i3}a_{i2}}{\left(b_{i1}+a_{i1}^{2}\right)\left(b_{i2}+a_{i2}^{2}\right)-{\left(b_{i3}+a_{i1}a_{i2}\right)}^{2}},\\ w_{i2}=\frac{b_{i1}a_{i2}-b_{i3}a_{i1}}{\left(b_{i1}+a_{i1}^{2}\right)\left(b_{i2}+a_{i2}^{2}\right)-{\left(b_{i3}+a_{i1}a_{i2}\right)}^{2}}, \end{array}$$

where *a*_*ij*_,  *b*_*ij*_, *j* = 1, 2 and *b*_*i*3_ are as above definitions, *i* = 1, 2, 3, 4. Hence, by applying the result in (9) to (6), a minimum value of $\text{MSE}({\hat{\sigma }}_{i})$ can be obtained as follows:

(10) MSE(σ^i)=bi1bi2−bi32(bi1+ai12)(bi2+ai22)−(bi3+ai1ai2)2,i=1,2,3,4,

where *a*_*ij*_, *b*_*ij*_, *j* = 1, 2 and *b*_*i*3_ are as above definitions, *i* = 1, 2, 3, 4 [12].

Based on (2) to (5), the estimators of location parameter *θ* for WMEs are computed by using moments method, and a weight is assigned in each equation from (11) to (14) as well. Thus, the estimators of location for WMEs are shown as follows:

(11) θ^1=w1[∑i=r+1r+kY(i)k−E11kσ^1]+(1−w1)×[∑i=r+k+l+1N−sY(i)N−s−r−k−l−E12N−s−r−k−lσ^1]=θ+σ[w1F11+(1−w1)F12],

(12) θ^2=w2[∑i=r+1r+kY(i)+lY(r+k)k+l−E21k+lσ^2]+(1−w2) ×[∑i=r+k+l+1N−sY(i)+sY(N−s)N−r−k−l−E22N−r−k−lσ^2]=θ+σ[w2F21+(1−w2)F22],

(13) θ^3=w3[∑i=r+1r+kY(i)k−E11kσ^3]+(1−w3)×[∑i=r+k+l+1N−sY(i)+sY(N−s)N−r−k−l−E22N−r−k−lσ^3]=θ+σ[w33F31+(1−w3)F32],

(14) θ^4=w4[∑i=r+1r+kY(i)+lY(r+k)k+l−E21k+lσ^4]+(1−w4)×[∑i=r+k+l+1N−sY(i)N−s−r−k−l−E12N−s−r−k−lσ^4]=θ+σ[w4F41+(1−w4)F42],

where ${\hat{\sigma }}_{1}$, ${\hat{\sigma }}_{2}$, ${\hat{\sigma }}_{3}$, and ${\hat{\sigma }}_{4}$ as (2) to (5), respectively,

(15) E11=E[∑i=r+1r+kX(i)],  E12=E[∑i=r+k+l+1N−sX(i)],E21=E[∑i=r+1r+kX(i)+lX(r+k)],E22=E[∑i=r+k+l+1N−sX(i)+sX(N−s)],F11=∑i=r+1r+kX(i)k−E11kσ^1σ,F12=∑i=r+k+l+1N−sX(i)N−s−r−k−l−E12N−s−r−k−lσ^1σ,F21=∑i=r+1r+kX(i)+lX(r+k)k+l−E21k+lσ^2σ,F22=∑i=r+k+l+1N−sX(i)+sX(N−s)N−r−k−l−E22N−r−k−lσ^2σ,F31=∑i=r+1r+kX(i)k−E11kσ^3σ,F32=∑i=r+k+l+1N−sX(i)+sX(N−s)N−r−k−l−E22N−r−k−lσ^3σ,F41=∑i=r+1r+kX(i)+lX(r+k)k+l−E21k+lσ^4σ,F42=∑i=r+k+l+1N−sX(i)N−s−r−k−l−E12N−s−r−k−lσ^4σ.

Similarly, the weights are computed by minimizing MSEs of ${\hat{\theta }}_{i}$ and the optimal weights in MSEs of ${\hat{\theta }}_{i}$ are obtained as

(16) wi=(Var⁡(Fi2)−Cov(Fi1,Fi2)−E(Fi1)E(Fi2)+[E(Fi2)]2) ×(Var⁡(Fi1)+Var⁡(Fi2)−2Cov(Fi1,Fi2) +[E(Fi1)]2−2E(Fi1)E(Fi2)+[E(Fi2)]2)−1.

The minimum value of MSEs of ${\hat{\theta }}_{i}$ can then be written as

(17) MSE(θ^i)=wi2{Var⁡(Fi1)+Var⁡(Fi2) −2Cov(Fi1,Fi2)+[E(Fi1)]2 −2E(Fi1)E(Fi2)+[E(Fi2)]2} −2wi{Var⁡(Fi2)−Cov(Fi1,Fi2)−E(Fi1)E(Fi2)+[E(Fi2)]2}+Var⁡(Fi2)+[E(Fi2)]2,

where *E*(*F*_*i*1_), *E*(*F*_*i*2_), Var⁡(*F*_*i*1_), Var⁡(*F*_*i*2_), and Cov(*F*_*i*1_, *F*_*i*2_) can be obtained based on (8) for *i* = 1, 2, 3, 4.

For the case of *l* = 0, the WMEs of the scale parameter *σ* are proposed as (18) since it is reasonable with consecutive data. Consider the following:

(18) σ^5=w5[∑i=r+2N−s(Y(i)−Y(r+1))]=w5C5,σ^6=w6[∑i=r+2N−s(Y(i)−Y(r+1))+s(Y(N−s)−Y(r+1))]=w6C6,σ^7=w7[r(Y(r+2)−Y(r+1))+∑i=r+2N−s(Y(i)−Y(r+1))]=w7C7,σ^8=w8[r(Y(r+2)−Y(r+1))+∑i=r+2N−s(Y(i)−Y(r+1)) +s(Y(N−s)−Y(r+1))]=w8C8.

In the same way, the minimum values of MSE of ${\hat{\sigma }}_{i}$, *i* = 5, 6, 7, 8 can be obtained as

(19) $$\begin{array}{c} \text{MSE}\left({\hat{\sigma }}_{i}\right)=\frac{a_{i}^{2}b_{i}+a_{i}^{4}}{{\left(b_{i}+a_{i}^{2}\right)}^{2}}-\frac{2a_{i}^{2}}{b_{i}+a_{i}^{2}}+1, \end{array}$$

where *a*_*i*_ = *E*(*C*_*i*_)/*σ* and *b*_*i*_ = Var⁡(*C*_*i*_)/*σ* ^2^, *i* = 5, 6, 7, 8. Thus, the WMEs ${\hat{\theta }}_{i}$ of the location parameter *θ* for the case of *l* = 0 can also be provided as follows:

(20) $$\begin{array}{c} {\hat{\theta }}_{5}=\frac{\sum_{i=r+1}^{N-s}Y_{\left(i\right)}}{N-s-r}-\frac{E_{5}}{N-s-r}{\hat{\sigma }}_{5},\\ {\hat{\theta }}_{6}=\frac{\sum_{i=r+1}^{N-s}Y_{\left(i\right)}+sY_{\left(N-s\right)}}{N-r}-\frac{E_{6}}{N-r}{\hat{\sigma }}_{6},\\ {\hat{\theta }}_{7}=\frac{rY_{\left(r+1\right)}+\sum_{i=r+1}^{N-s}Y_{\left(i\right)}}{N-s}-\frac{E_{7}}{N-s}{\hat{\sigma }}_{7},\\ {\hat{\theta }}_{8}=\frac{rY_{\left(r+1\right)}+\sum_{i=r+1}^{N-s}Y_{\left(i\right)}+sY_{\left(N-s\right)}}{N}-\frac{E_{8}}{N}{\hat{\sigma }}_{8}, \end{array}$$

where  *E*_5_ = *E*[∑_*i*=*r*+1_^*N*−*s*^ *X*_(*i*)_], *E*_6_ = *E*[∑_*i*=*r*+1_^*N*−*s*^ *X*_(*i*)_ + *sX*_(*N*−*s*)_], *E*_7_ = *E*[*rX*_(*r*+1)_ + ∑_*i*=*r*+1_^*N*−*s*^ *X*_(*i*)_], *E*_8_ = *E*[*rX*_(*r*+1)_ + ∑_*i*=*r*+1_^*N*−*s*^ *X*_(*i*)_ + *sX*_(*N*−*s*)_], and ${\hat{\sigma }}_{5}$, ${\hat{\sigma }}_{6}$, ${\hat{\sigma }}_{7}$, ${\hat{\sigma }}_{8}$ as (18). The MSEs of ${\hat{\theta }}_{i}$ are obtained by using $\text{MSE}({\hat{\theta }}_{i})=\mathrm{Var}({\hat{\theta }}_{i})+{\left[E({\hat{\theta }}_{i})-\theta \right]}^{2}$ for *i* = 5, 6, 7, 8.

Next, we extend the method of Balakrishnan and Cohen [13] and Balakrishnan and Chan [4] to derive the BLUE of the location and scale parameters in an explicit form under a multiply type II censored sample for the general case. BLUE ${\hat{\theta }}_{9}$ and ${\hat{\sigma }}_{9}$ are obtained as follows:

(21) $$\begin{array}{c} {\hat{\theta }}_{9}=\left\{\frac{\alpha^{T}\beta^{-1}\alpha 1^{T}\beta^{-1}-\alpha^{T}\beta^{-1}1\alpha^{T}\beta^{-1}}{\left(\alpha^{T}\beta^{-1}\alpha \right)\left(1^{T}\beta^{-1}1\right)-{\left(\alpha^{T}\beta^{-1}1\right)}^{2}}\right\}Y,\\ {\hat{\sigma }}_{9}=\left\{\frac{1^{T}\beta^{-1}1\alpha^{T}\beta^{-1}-1^{T}\beta^{-1}\alpha 1^{T}\beta^{-1}}{\left(\alpha^{T}\beta^{-1}\alpha \right)\left(1^{T}\beta^{-1}1\right)-{\left(\alpha^{T}\beta^{-1}1\right)}^{2}}\right\}Y, \end{array}$$

where

(22) 1=(1,1,…,1,1,…,1)(N−s−r−l)×1TY=(Y(r+1),Y(r+2),…,Y(r+k),Y(r+k+l+1),Y(r+k+l+2),…,Y(N−s))Tα=(α(r+1),α(r+2),…,α(r+k),α(r+k+l+1),α(r+k+l+2),…,α(N−s)),β=[β(r+1,r+1)β(r+1,r+2)⋯β(r+1,r+k)β(r+1,r+k+l+1)⋯    β(r+1,N−s)β(r+1,r+2)β(r+2,r+2)⋯  β(r+2,r+k)β(r+1,r+k+l+1)⋯β(r+2,N−s)⋮⋮⋱⋮⋮⋱⋮β(r+1,r+k)β(r+2,r+k)⋯  β(r+k,r+k)β(r+1,r+k+l+1)⋯β(r+k,N−s)β(r+1,r+k+l+1)β(r+2,r+k+l+1)⋯β(r+k,r+k+l+1)    β(r+1,r+k+l+1)⋯  β(r+k+l+1,N−s)β(r+1,r+k+l+2)  β(r+2,r+k+l+2)⋯β(r+k,r+k+l+2)β(r+1,r+k+l+1)⋯β(r+k+l+2,N−s)⋮⋮⋱⋮⋮⋱⋮β(r+1,N−s)β(r+2,N−s)⋯β(r+k,N−s)β(r+1,r+k+l+1)⋯β(N−s,N−s)],αi=E(Y(i)−θσ)=E(X(i)),β(i,j)=Cov(Y(i)−θσ,Y(j)−θσ)=Cov(X(i),X(j)).

*E*(*X*_(*i*)_) and Cov(*X*_(*i*)_, *X*_(*j*)_) are as above definition.

Further, the MSEs of BLUE ${\hat{\theta }}_{9}$ and ${\hat{\sigma }}_{9}$ are given by

(23) MSE(θ^9)=Var⁡(θ^9)=σ2{αTβ−1α(αTβ−1α)(1Tβ−11)−(αTβ−11)2},MSE(σ^9)=Var⁡(σ^9)=σ2{1Tβ−11(αTβ−1α)(1Tβ−11)−(αTβ−11)2},

where 1, *α* and *β* are as above definition. In addition, we find the best estimators based on the smallest MSEs among the WMEs and BLUE, respectively, as follows:

(24) MSE1≤i≤9(θ^i),  MSE1≤i≤9(σ^i).

## 3. The Comparative Numerical Results

To study the behavior of our proposed WMEs and BLUE, the simulated MSEs of BLUE and exact MSEs of WMEs are compared. The numerical results were obtained to cover different censoring schemes assigning appropriate values to *r*, *k*, *l*, and *s* for sample size *N* = 15, 25, *σ* = 1, and *θ* = 1. The study results are summarized in Tables 1 to 4.

For comparison results of scale estimators for *σ*, Tables 1 and 2 indicate that (i) WME2 is the best estimator for *N* = 15, *l* ≠ 0, and all different censoring schemes except (*r*, *k*, *l*, *s*) = (2,5, 3,1), (1,5, 2,3), (1,3, 7,1), (1,5, 1,4); (ii) WME3 is the best estimator for *N* = 25, *l* ≠ 0, and all different censoring schemes except (*r*, *k*, *l*, *s*) = (3,5, 2,1),  (3,5, 1,2), (1,3, 7,1); (iii) WME8 is the best estimator for *N* = 15,25 and right censoring scheme; (iv) WME5(=WME6) is the best estimator for *N* = 15,25 and left censoring scheme; (v) WME6 is the best estimator for *N* = 15,25 and left censoring scheme. The magnitude of MSE decreases as sample size *N* increases. The value of the smallest MSE divided by BLUE is denoted as WME/BLUE, and its range is between 0.821384 and 0.996789 for *N* = 15,25 and *l* ≠ 0. It means that our smallest MSE of WMEs is smaller than that of BLUE. The range of WME/BLUE is between 0.794971 and 0.987798 for *N* = 15,25 and *l* = 0. It means that our smallest MSE of WMEs is smaller than that of BLUE.

For comparison results of location estimators for *θ*, Tables 3 and 4 show that (i) WME1 is the best estimator for *N* = 15, (*r*, *k*, *l*, *s*) = (1, 5, 3, 2), (1, 5, 2, 3), (1, 3, 7, 1), (1, 5, 1, 4), and middle censoring scheme; (ii) WME1 is the best estimator for *N* = 25 and all different censoring schemes except right censoring scheme; (iii) WME8 is the best estimator for *N* = 15 and right censoring scheme; (iv) WME5 is the best estimator for *N* = 25 and left and doubly censoring schemes. The magnitude of MSE decreases as sample size *N* increases. The range of WME/BLUE is between 0.861855 and 1.048036 for *N* = 15,25 and *l* ≠ 0. The range of WME/BLUE is between 0.843873 and 1.02405 for *N* = 15,25 and *l* = 0.

## 4. Conclusion

The object of this paper is to propose WMEs of location parameter *θ* and scale parameter *σ* of the extreme value distribution based on the multiply type II censored sample. The comparison results of scale estimators find that our proposed WMEs are, in general, superior to BLUE under different combinations of censoring schemes and sample size. On the other hand, the comparison results of location estimators present that for *N* = 15, our proposed WMEs are, in general, superior to BLUE and different censoring schemes except (*r*, *k*, *l*, *s*) = (2, 5, 2, 2), (3, 5, 2, 1), (3, 5, 1, 2), (2, 5, 3, 1), (2, 5, 1, 3), (4, 5, 1, 1), left and doubly censoring schemes; for *N* = 25 our proposed WMEs are, in general, superior to BLUE and all different censoring schemes except right censoring scheme.

WMEs and BLUE can further be used to predict the *j*th (*N* − *s* < *j* ≤ *N*) ordered observation *Y*_(*j*)_ in a sample of size *n* from the extreme value distribution based on multiply type II censored sample. Moreover, if observations *T* = exp⁡(−*Y*) are from a Weibull distribution with a scale parameter *α* and a shape parameter *β*, then the best estimators of Weibull distribution can be obtained through proposing the best estimators of the extreme value distribution, where *α* = exp⁡(−*θ*) and *β* = 1/*σ*. Moreover, by replacing *Y* by −*Y*, the corresponding distribution of −*Y* is also called extreme value distribution with c.d.f. as given by

(25) $$\begin{array}{c} F_{-Y}\left(y\right)=1-\exp\left[-e^{(y-\mu )/\sigma }\right],\;-\infty <y<\infty , \end{array}$$

where −*∞* < *μ* = −*θ* < *∞*, *σ* > 0. Our method and the best linear unbiased method can be used to find the best estimator of *μ* and *σ* and predict the *j*th (*N* − *s* < *j* ≤ *N*) ordered observation *Y*_(*j*)_ for extreme value distribution with c.d.f. as (25) based on negative transformation and the multiply type II censored sample.

## Figures and Tables

**Table 1:** MSEs of BLUE and WMEs of the scale parameter *σ* at *N* = 15, *σ* = 1.0, and θ = 1.

Censoring scheme type II	*r*	*k*	*l*	*s*	The simulated MSEs of BLUE and exact MSEs of WMEs					
					BLUE	WME1	WME2	WME3	WME4	WME/BLUE
Multiply	2	5	2	2	0.070396	0.067245	0.066049*	0.066182	0.067211	0.938249
	3	5	2	1	0.074345	0.069957	0.069488*	0.069732	0.069821	0.934669
	3	5	1	2	0.081776	0.077573	0.075709*	0.075784	0.077564	0.925810
	2	5	3	1	0.064955	0.061733	0.061551	0.061859	0.061543*	0.947471
	2	5	1	3	0.077774	0.074938	0.072323*	0.072350	0.074945	0.929912
	1	5	3	2	0.060664	0.058404	0.057795*	0.057900	0.058413	0.952707
	1	5	2	3	0.066040	0.064033	0.062353	0.062348*	0.064107	0.944094
	4	5	1	1	0.085554	0.079741	0.078920*	0.079045	0.079696	0.922458
	1	3	7	1	0.059028	0.056334	0.057378	0.057511	0.056253*	0.952984
	1	5	1	4	0.073128	0.071480	0.068433	0.068391*	0.071521	0.935223
Middle	0	3	6	0	0.045595	0.044507*	0.044635	0.044507*	0.044635	0.976138

					BLUE	WME5	WME6	WME7	WME8	WME/BLUE

Right	0	0	0	8	0.101814	0.132434	0.102793	0.132434	0.084350*	0.828472
Left	8	3	0	0	0.145212	0.127135*	0.127135*	0.349345	0.349345	0.875513
Doubly	7	3	0	2	0.169211	0.155749	0.145217*	0.477876	0.300771	0.858200

*The smallest MSE among the proposed WMEs and BLUE.

**Table 2:** MSEs of BLUE and WMEs of the scale parameter *σ* at *N* = 25, *σ* = 1.0, and θ = 1.

Censoring scheme type II	*r*	*k*	*l*	*s*	The simulated MSEs of BLUE and exact MSEs of WMEs					
					BLUE	WME1	WME2	WME3	WME4	WME/BLUE
Multiply	2	5	2	2	0.033049	0.033973	0.032609	0.032605*	0.033950	0.986562
	3	5	2	1	0.034131	0.034709	0.034021*	0.034042	0.034667	0.996789
	3	5	1	2	0.036422	0.037066	0.035446*	0.035476	0.036991	0.973205
	2	5	3	1	0.038106	0.031840	0.031332	0.031300*	0.031862	0.821384
	2	5	1	3	0.034793	0.036526	0.034272	0.034250*	0.036474	0.984403
	1	5	3	2	0.030754	0.031121	0.030042	0.029959*	0.031178	0.974163
	1	5	2	3	0.031932	0.033203	0.031209	0.031211*	0.033194	0.977406
	4	5	1	1	0.041570	0.037365	0.036608	0.036605*	0.037358	0.880563
	1	3	7	1	0.030205	0.029124	0.028682*	0.028727	0.029052	0.949578
	1	5	1	4	0.033436	0.035586	0.032923	0.032814*	0.035537	0.981404
Middle	0	3	6	0	0.026008	0.025694*	0.025757	0.025694*	0.025757	0.987927

					BLUE	WME5	WME6	WME7	WME8	WME/BLUE

Right	0	0	0	8	0.038026	0.058844	0.046303	0.058844	0.037562*	0.987798
Left	8	3	0	0	0.060523	0.048114*	0.048114*	0.069671	0.069671	0.794971
Doubly	7	3	0	2	0.056175	0.053227	0.049471*	0.09685	0.064317	0.880659

*The smallest MSE among the proposed WMEs and BLUE.

**Table 3:** MSEs of BLUE and WMEs of the location parameter *θ* at *N* = 15, *σ* = 1.0, and θ = 1.

Censoring scheme type II	*r*	*k*	*l*	*s*	The simulated MSEs of BLUE and exact MSEs of WMEs					
					BLUE	WME1	WME2	WME3	WME4	WME/BLUE
Multiply	2	5	2	2	0.081471*	0.084486	0.088129	0.087748	0.089356	1.037007
	3	5	2	1	0.087399*	0.093767	0.097813	0.091590	0.098849	1.047952
	3	5	1	2	0.087310*	0.093792	0.094834	0.091504	0.096280	1.048036
	2	5	3	1	0.081571*	0.084650	0.090575	0.085397	0.091483	1.037746
	2	5	1	3	0.081668*	0.084633	0.086168	0.090419	0.087180	1.036306
	1	5	3	2	0.078327	0.076445*	0.083378	0.085178	0.083145	0.975973
	1	5	2	3	0.078619	0.076260*	0.081964	0.089104	0.081176	0.969995
	4	5	1	1	0.097020*	0.105156	0.107617	0.100723	0.107746	1.038167
	1	3	7	1	0.083615	0.076417*	0.089073	0.089757	0.086154	0.913925
	1	5	1	4	0.079463	0.076634*	0.081216	0.093448	0.079339	0.964399
Middle	0	3	6	0	0.075444	0.071020*	0.079400	0.084294	0.079400	0.941360

					BLUE	WME5	WME6	WME7	WME8	WME/BLUE

Right	0	0	0	8	0.095452	0.105177	0.094909	0.105177	0.094913*	0.99431
Left	8	3	0	0	0.228809*	0.234311	0.234311	0.463530	0.463530	1.02405
Doubly	7	3	0	2	0.182548*	0.184806	0.186032	0.310332	0.268930	1.01237

*The smallest MSE among the proposed WMEs and BLUE.

**Table 4:** MSEs of BLUE and WMEs of the location parameter *θ* at *N* = 25, *σ* = 1.0, and θ = 1.

Censoring scheme type II	*r*	*k*	*l*	*s*	The simulated MSEs of BLUE and exact MSEs of WMEs					
					BLUE	WME1	WME2	WME3	WME4	WME/BLUE
Multiply	2	5	2	2	0.047455	0.043392*	0.047048	0.046523	0.045351	0.914382
	3	5	2	1	0.047910	0.046756*	0.049381	0.047447	0.048703	0.975913
	3	5	1	2	0.048516	0.046415*	0.048644	0.047332	0.047459	0.956695
	2	5	3	1	0.048501	0.043917*	0.047599	0.046681	0.046598	0.905486
	2	5	1	3	0.046475	0.043009*	0.046365	0.046338	0.044072	0.925422
	1	5	3	2	0.046091	0.040401*	0.045429	0.046284	0.043109	0.876549
	1	5	2	3	0.045630	0.039876*	0.045084	0.045986	0.041846	0.873899
	4	5	1	1	0.051705	0.050038	0.051508	0.048829*	0.051088	0.944386
	1	3	7	1	0.047423	0.041238*	0.047731	0.050815	0.045522	0.861855
	1	5	1	4	0.045836	0.039504*	0.044303	0.045540	0.040560	0.861855
Middle	0	3	6	0	0.043759	0.039707*	0.043286	0.052436	0.043286	0.907402

					BLUE	WME5	WME6	WME7	WME8	WME/BLUE

Right	0	0	0	8	0.047960*	0.048812	0.049585	0.048812	0.04907	1.017765
Left	8	3	0	0	0.078955	0.066628*	0.066628*	0.076515	0.076515	0.843873
Doubly	7	3	0	2	0.062959	0.060392*	0.061361	0.068265	0.064084	0.959227

*The smallest MSE among the proposed WMEs and BLUE.
